# Isolation by distance and non-identical patterns of gene flow within two river populations of the freshwater fish *Rutilus rutilus* (L. 1758)

**DOI:** 10.1007/s10592-016-0828-3

**Published:** 2016-03-10

**Authors:** S. Crookes, P. W. Shaw

**Affiliations:** 1 Great Lakes Institute for Environmental Research (GLIER), University of Windsor, 401 Sunset Avenue, Windsor, ON N9B 3P4 Canada; 2 Aquatic, Behavioural, & Evolutionary Biology Research Group, Institute of Biological, Environmental and Rural Sciences, University of Aberystwyth, Aberystwyth, Ceredigion SY23 3DA UK; 3 School of Biological Sciences, Centre for Ecology, Evolution and Behaviour, Royal Holloway, University of London, Egham, Surrey, TW20 0EX UK

**Keywords:** IBD, Microsatellite, Fisheries, Connectivity, *N*_e_

## Abstract

The spatial distribution of organisms is maintained by a combination of in situ reproduction and dispersal of conspecifics from elsewhere within its habitable range. The determination of dispersal origin and sub-population connectivity has a vital role to play in forming effective management policies. The common roach (*Rutilus rutilus*) is an important component of the economically and socially valuable recreational fishery and represents a well-studied member of the Cyprinidae. Microsatellite allele data were used to investigate hypothetically variant levels of microevolutionary structuring and isolation-by-distance (IBD) in in the Rivers Stour and Thames. A strong signal of IBD was found in the Stour, probably due to the limited capacity for unrestricted bidirectional dispersal in this river compared with the Thames. A weak inference of IBD in the Thames is likely erroneous and effected by a strong localised genetic signal from a recent stocking event. Whilst we found significantly genetically divergent upstream areas in the River Stour, a strong signal of IBD remained when the headwater sub-population was removed, suggesting that that the signal is not biased by non-equilibrium conditions in upstream reaches. We discuss these results with reference to the management of aquatic bioresources and emphasise the idiosyncrasy that aquatic biota and hydrological complexity may imprint upon patterns of biodiversity within any given system.

**Electronic supplementary material:**

The online version of this article (doi:10.1007/s10592-016-0828-3) contains supplementary material, which is available to authorized users.

## Introduction

The ability to determine the extent to which sub-populations are connected is vital to understanding, conserving and managing populations (Hughes et al. 2009). Migration provides a means other than direct recruitment for an organism to maintain temporal and spatial persistence. Most species display some degree of population sub-structuring, dependent upon physical limitations of habitats and the capability for dispersal. Gene flow is essential to negate the potentially deleterious impact of inbreeding and to maintain variation to maximise adaptive potential (Frankham 1996). In lentic environments, rivers channel the movements of aquatic organisms along physically delimited pathways resulting in a directional bias to passive dispersal (Fagan 2002). Riverine ecosystems consist of a patchwork of habitat (Matthews 1998), the distribution of which may vary due to the effects of periodic droughts or floods. Such events may facilitate or impede the ability of individuals to commute. Moreover, anthropogenic modification may drastically alter the natural state of riverine ecosystems (e.g., Bravard et al. 1986) potentially obstructing free passage. Impeded gene flow may lead to significant genetic structuring within a system. By measuring the extent to which populations are genetically sub-structured, researchers can infer the degree to which sub-populations are connected through effective migration, a parameter vital to the implementation of cohesive strategies to manage biodiversity.

Isolation by distance (IBD) (Wright 1946) describes the linear relationship between genetic differentiation and geographical distance. In rivers, the most intuitive equilibrium model of population structure is the stepping-stone model (SSM: Kimura and Weiss 1964), within which a directional distance-correlation function is applied to the probability of migrant exchange. However, the strong influence of unidirectional water flow may impede IBD, isolating headwater populations, leading to demographic bottlenecking and/or localised extinctions and re-colonisation (non-equilibrium conditions) (Fraser et al. 2004; Hänfling and Weetman 2006). Identifying IBD is important to determine the ability and extent to which gene flow may replenish neighbouring areas in the event of localised extinctions. Equally valuable to managers is the identification of areas under non-equilibrium conditions, which may contain unique genotypic combinations (Wade and McCauley 1988). By discerning the competing influences of migration and genetic drift from neutral genetic data one may be able to determine the relative support for equilibrium or non-equilibrium scenarios in any given area (Hutchinson and Templeton 1999).

Intraspecific gene flow in aquatic species is amenable to anthropogenic influences. Arterial canals and bank modification may facilitate the long-distance dispersal of fishes, potentially homogenising genetically divergent populations (Lynch et al. 2011). Further, anthropogenic constructions such as dams, gauging weirs, mills and disused locks may pose significant obstacles for upstream migration (Lucas and Frear 1997; Geeraerts et al. 2007). Moreover, the abstraction of water for anthropogenic use has the potential to periodically close-off upstream reaches from downstream sub-populations (Fischer and Kummer 2000). The Thames and Stour in southeast England represent two rivers with variant natural and modified hydrologies. Whilst both have a history of modification originating before the Industrial Revolution, the use of the River Stour for large-scale shipping ended in the mid- nineteenth century with only 24 km made available for navigation compared to over 300 km in the Thames. However, the Stour retains many weirs, disused locks and mills that likely obstruct the upstream movements of fishes. It is a hypothetical possibility that routes of upstream migration in the Thames are more porous due to continual heavy use of this waterway by industrial and civil vessels.

Both the Thames and the Stour contain a coarse fishery which represents a significant component of the socio-economic makeup of developed nations (Weithmann 1999). In the UK, the eurytopic roach (*Rutilus rutilus*) is a keystone species for the angling community (Robinson et al. 2003) and is found across lowland systems. Its abundance, motility and potential for natal philopatry during spawning (Goldspink 1977; L’Abée-Lund and Vøllestad 1985), thereby exacerbating genetic structuring (Massicotte et al. 2008), make it an excellent candidate model species to investigate patterns of gene flow. Whilst Hamilton et al. (2014) found evidence for IBD in roach in the Thames, it was only on a system-wide scale; they did not specifically focus on the issue of connectivity within linear stretches of river. By contrast, we investigate the distribution of microsatellite variation along the Rivers Stour and Thames to determine the effects of these river’s traits upon levels of IBD and gene flow. Explicitly, we hypothesise connectivity will be lower within the Stour than within the Thames, with concomitantly higher levels of genetic differentiation. Furthermore, if there is significant obstruction to migration in the Stour, we would predict strong IBD over short distances but at larger spatial scales the signal would be lost through allelic differences at independent loci accruing through genetic drift (Type IV IBD sensu Hutchinson and Templeton 1999). We also predict an increase in population inbreeding in the Stour relative to that found in the Thames, manifesting in lower effective sub-population sizes, seeking to corroborate the low sub-population effective population sizes (*N*_e_s) reported in this species (Hamilton et al. 2014). Finally, we seek to observe whether there exists a spike in genetic divergence at a site within the Thames that has a history of large-scale stocking.

## Methods

### Field sampling

**Fig. 1:**
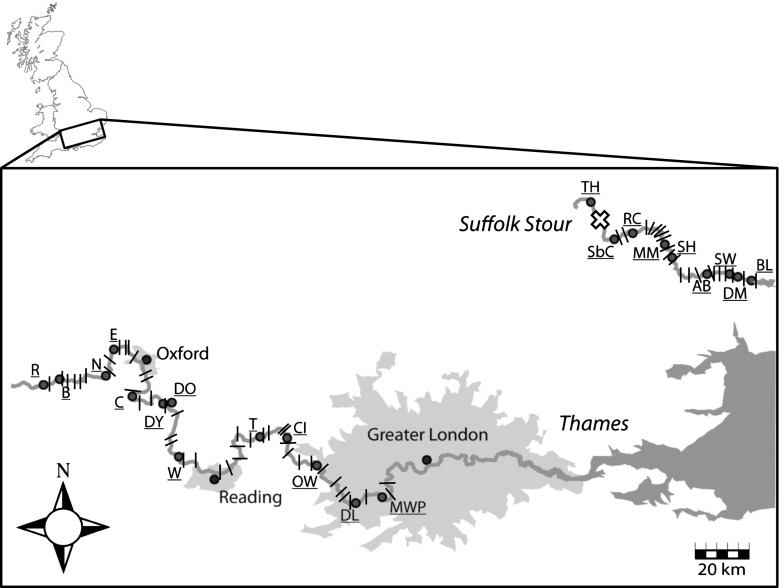
Map conveying the sampling locations (*dots*) within the Rivers Thames and Suffolk Stour in Southeast England (see Table 1 for codes). Also shown are the locations of weirs and locks (*dashes*) that may impede free passage of freshwater fishes in the upstream direction. The cross symbolises a major water extraction point in the Stour

The Environment Agency of England and Wales (EA) (Table 1; Fig. 1) sampled a total of 1001 cycloid scales from individual roach during the annual electrofishing surveys of Summer/Autumn of 2006. 507 individuals were collected from thirteen locations distributed along 185 km of the main River Thames, along with a further 494 from nine locations distributed along almost the entire length of the River Stour (66 km). All fish were sampled in 100 m segments of river immediately downstream from an anthropogenic feature, usually a weir. Each scale was air dried, sealed in an envelope and stored until further analysis.

**Table 1:** Sampling information and geographic and genetic diversity metrics (observed and expected heterozygosity (H_o_ and H_e_, respectively), Allelic richness (A_r_) and the number of alleles (NA)) for each of the 22 study locations used in this study

Sampling site	Code	Geographical co-ordinates		Sample Size	Microsatellite variation			
		Latitude	Longitude		*H*_o_	*H*_e_	A_r_	NA
Thames								
Molesey Weir Pool	MWP	51.405637	−0.345167	33	0.633	0.700	8.592	10.400
Desborough Loop	DL	51.383549	−0.439374	65	0.644	0.732	8.645	11.900
Old Windsor	OW	51.485767	−0.589391	49	0.656	0.724	8.473	11.200
Clivedon Island	CI	51.545816	−0.693418	63	0.630	0.732	8.600	12.200
Temple	T	51.551675	−0.792194	60	0.674	0.719	8.675	12.100
Whitchurch	W	51.486617	−1.089,740	23	0.643	0.738	8.534	9.500
Dorchester	DO	51.641830	−1.164674	33	0.637	0.750	8.133	9.600
Days	DY	51.638345	−1.180634	33	0.570	0.717	8.036	9.700
Culham	C	51.646155	−1.274436	40	0.659	0.723	8.223	10.200
Eynsham^a^	E	51.775208	−1.356433	33	0.602	0.703	8.463	10.300
Northmoor	N	51.716871	−1.376079	26	0.628	0.724	8.533	9.600
Buscot	B	51.681196	−1.668736	27	0.617	0.688	8.454	9.778
Roundhouse	R	51.686687	−1.704859	22	0.659	0.733	8.475	9.300
Mean				39	0.635	0.722	8.449	10.444
Suffolk Stour								
Brantham Lock	BL	51.956858	1.040325	86	0.599	0.682	6.716	11.500
Dedham Mill	DM	51.963634	0.995650	40	0.601	0.687	6.411	8.500
Stratford Weir	SW	51.961420	0.976577	84	0.606	0.720	7.271	11.700
Anchor Bridge	AB	51.968510	0.872212	59	0.582	0.764	8.244	14.900
Shalford Weir	SH	52.007552	0.743557	64	0.649	0.672	6.662	9.000
Mill Meadow	MM	52.038694	0.719216	78	0.611	0.705	6.992	11.000
Rat’s Castle	RC	52.078182	0.603393	20	0.630	0.747	7.116	7.889
Stoke-by-Clare^a^	SbC	52.057994	0.539488	43	0.570	0.707	6.736	9.100
Thurlow	TH	52.122522	0.455658	20	0.606	0.688	6.672	7.900
Mean				55	0.606	0.708	6.980	10.165

^a^Represent pooled samples of roach from two adjacent sites to increase sample size. All diversity indices are derived from analyses of microsatellite length variation at ten independently inherited loci (see Table 2)

### Study system

Historically both rivers were tributaries of the English Channel River system that drained most of Western Europe. They were sundered approximately 7500 years ago upon flooding of the English Channel. Recently, the EA’s River Habitat Surveys (RHS) reported that 60 % of sites along the Thames were significantly or severely modified, with only 7 % of sites described as ‘pristine’ (Johnson et al. 2009). However, efforts are ongoing to continue riverine habitat restoration that has gathered momentum during the last 30 years. These efforts include stocking fish to maintain recreational fisheries. This study includes a site (MWP) situated just upstream of a large introduction of 45,000 juvenile roach some 6 years prior. Similarly, the Suffolk Stour also has a history of modification, albeit much less so than the Thames. There are 45 and 21 potentially significant barriers to dispersal (e.g., a weir, lock or mill) between the most upstream and downstream sample sites in the Thames and Stour, respectively (Fig. 1). Although most locks are still in working order in the Thames, only those downstream of Shalford Weir inclusively currently operate in the Stour.

### Microsatellite diversity

DNA was extracted from scales using the CTAB method (Winnepennickx et al. 1993). All loci and their amplifying primers were mined from the literature (Table 2). PCR products were visualised on 6 % acrylamide gels. All genotyping was performed using ALF express II and III™ (Amersham Pharmacia Biotech, UK) automated sequencers with molecular ladders of known size. Proprietary software (Fragment Manager version 1.2) was employed to genotype individuals at all loci. In order to assess microsatellite diversity in sub-populations from which estimates of IBD and other genetic parameters can be calculated, both bias-corrected (H_e_) and uncorrected (H_o_) estimates of heterozygosity (Nei 1973), the number of alleles, and allelic richness were calculated for all individual locations, rivers, and for the global dataset in the program FSTAT version 2.9.3 (Goudet 1995). Mann–Whitney U-tests were employed to differentiate between means if normality was not met. The influence of null alleles was determined in FreeNA (Chapuis and Estoup 2007) by computing the genetic divergence parameter *F*_ST_ after accounting for null allele frequency (frequencies of ≥0.2 are considered large). The dataset was permuted 1000 times to determine statistical significance and 95 % confidence intervals (95 % CIs).

**Table 2:** Information regarding microsatellite loci utilised in the present study

Locus	Repeat motif	Original publication	Number of alleles	Size range of PCR products^d^
Rru3	(ACTC)_5_N_21_(GT)_7_A(TG)_6_	Barinova et al. (2004)	4^a^; 5^b^; 14^d^	173–183
Lid1	(CT)_5_(CA)_20_	Barinova et al. (2004)	4^a^; 8^b^; 15^d^	226–248
CypG3	(CAGA)_2_(TAGA)_11_	Baerwald and May (2004)	16^b^; 37^d^	194–338
CypG48	(TAGA)_8_TACGG(TAGA)_10_	Baerwald and May (2004)	22^d^	168–236
Ca1	(CA)_24_	Dimsoski et al. (2000)	7^b^; 20^d^	104–138
Ca3	(TAGA)_14_	Dimsoski et al. (2000)	18^b^; 32^d^	236–312
Ca12	(TAGA)_10_(CAGA)_4_(TAGA)_2_	Dimsoski et al. (2000)	30^d^	163–259
Lc27	(CT)_22_(CACT)_3_(CT)_2_	Vyskočilova et al. (2007)	3^c^; 8^d^	139–151
Lc290	(GA)_4_N_49_(CT)_13_TT(CT)_15_CC(CT)_2_CC(CT)_11_CC(CT)_3_	Vyskočilova et al. (2007)	6^c^; 17^d^	177–197
Lco4	(GT)_5_ATTTT(GT)_5_(GA)_11_	Turner et al. (2004)	2^c^; 10^d^	226–234

^a^Barinova et al. (2004); ^b^ Hamilton and Tyler (2008); ^c^ Vyskočilova et al. (2007); ^d^ Present study

To detect deviations from Hardy–Weinberg equilibrium (HWE), and to determine linkage disequilibrium between pairs of loci, in all sub-populations, Fisher’s exact tests (Raymond and Rousset 1995) were employed in Genepop version 4.0 (Rousset 2008). The randomised sampling procedure (Markov Chain Monte Carlo (MCMC)) was iterated 5000 times per 10^3^ batches, of which 10^5^ steps were discarded as ‘burn-in’. The log likelihood ratio statistic G (Guo and Thompson 1992) was used to detect deviations from the null expectation of HWE. The conservative Bonferroni correction (Rice 1989) was applied across all co-estimated results.

### Population structure and isolation by distance

We assessed pairwise genetic differentiation among sub-populations within rivers by application of Weir and Cockerham’s (1984) unbiased estimator of *F*_ST_ (θ), and Fisher’s exact tests of allelic differentiation in FSTAT and Genepop, respectively. 95 % CIs for all estimates of pairwise *F*_ST_ were calculated by jackknifing the dataset 1000 times. We assessed levels of hierarchical structuring across all sub-populations by conducting an AMOVA analysis in the program GENODIVE (Meirmans and van Tienderen 2004). The data were permuted 1000 times. We also applied a two-fold principal component analysis (PCA), analysing both allelic (*F*_ST_) and genotypic frequencies (co-dominant genotypic distance (CGD)) separately in GenAlEx version 6.5 (Peakall and Smouse 2012). Statistical significance was assessed by permuting the data 999 times.

In order to identify IBD, and to discover which environmental distance variables may influence the distribution of genetic variation among sub-populations, we employed both simple and partial Mantel tests implemented in IBD version 3.15 (Jensen et al. 2005), whereby genetic distance, river distance and environmental data were subject to a reduced major axis regression. Environmental data included the numbers of gauging weirs and tributaries between sites, respectively, concentration of the endocrine disrupting chemical (EDC) oestrone (ng L^−1^), and flow rate (m^3^ s^−1^). Oestrone concentrations were derived from data collected by the Centre for Ecology and Hydrology, UK, for all rivers in England and Wales, whereas flow rates were derived from the Environmental Agency’s network of flow gauging weirs. EDCs are suspected to increase between-population divergence by increasing reproductive variance at sites with high concentrations through the production of infertile intersex males (Harris et al. 2011). These variables were regressed against genetic divergence whilst controlling for river distance. Although Mantel tests have come under recent criticism over issues related to type I errors associated with autocorrelation of data points and overestimation of statistical significance (Guillot and Rousset 2013), these tests may still be powerful approaches to spatial genetic analysis when data are independent and assumptions of normality are met (Diniz-Filho et al. 2013). Data matrices were permuted 10^6^ times. Genetic distances were inputted as either *F*_ST_ or converted to the linearised form, *F*_ST_/(1−*F*_ST_), prior to the analysis. River distances were all derived from hydrologic data collected by Moore et al. (1994) and implemented in ArcGIS 9.4.

### Estimation of *N*_e_ and bottlenecking

So as to estimate effective sizes of local sub-populations, we performed a sibship assignment analysis in COLONY version 2.0 (Jones and Wang 2009). This method uses a maximum likelihood procedure to co-estimate both demographic (proportion of full and half-sib dyads) and genetic parameters, from which point estimates of *N*_e_ are calculated. Because the estimation of *N*_e_ of a sub-population may be upwardly biased by the inclusion of direct immigrants, but not by the descendants of immigrants (Wang 2009), putative first generation migrants were identified in the program GeneClass 2.0 (Piry et al. 2004), using the Bayesian method of Rannala and Mountain (1997), and discarded from the analysis. To detect recent reductions in genetic diversity, the program BOTTLENECK (Piry et al. 1999) was used applying coalescent simulations to derive statistical distributions of expected and observed heterozygosities under both equilibrium and non-equilibrium conditions. We assumed a two-phase model of microsatellite evolution, whereby most mutations follow the stepwise mutation model but a set proportion follow the infinite allele model (IAM). The proportion of IAM events in a two-phase model was set to 12 % following Garza and Williamson (2001). Significance was determined using Wilcoxon-signed rank tests.

### Population connectivity and gene flow

We estimated contemporary gene flow among sub-populations using a Bayesian method implemented in BAYESASS + version 3.0 (Wilson and Rannala 2003). Short MCMC runs were conducted to ascertain delta values that best maximise the most optimal acceptance ratios for implementation in the long runs from which final migration rates were extrapolated. The output files of each run were assessed for stationarity in Tracer version 1.6 (Rambaut and Drummond 2007). Analyses were iterated 5 times and mean values tabulated. Additionally, an autocorrelation analysis was undertaken in GenAlEx (Smouse and Peakall 1999) to determine the distance at which the genetic similarity between individuals becomes uncorrelated. This analysis calculates the correlation coefficient, *r*, of allelic diversity between individuals at increasing spatial scales. Individual genetic distances were calculated via the squared distances statistic φ_PT_ (Peakall et al. 2003), with all individuals from the same location recorded as belonging to the same distance category. *r* was then calculated as a function of seven discrete distance classes (km) for the Thames sub-populations: 5, 10, 20, 30, 60, 80 and 100; and as a function of five classes for the Stour sub-populations: 5, 10, 20, 30 and 60. 95 % CIs around point estimates of *r*, and either side of the null hypothesis of zero genetic structure, were calculated by 999 bootstrap iterations.

## Results

### Genetic diversity

All loci were found to be in linkage equilibrium. The FreeNA analysis found that the relationship between *F*_ST_ and corrected *F*_ST_ approached linearity (R^2^ = 0.9948, *p* ≪ 0.001), indicating a negligible influence of null alleles. Of the 129 locus-by-location comparisons in the Thames (Table S1), ten were found to violate the assumptions of HWE, close to the 6.45 expected by chance. This number was greater in the Stour, where 20 out of 88 locus-by-location comparisons all showed a deficit of heterozygotes (Table S2).

**Fig. 2:**
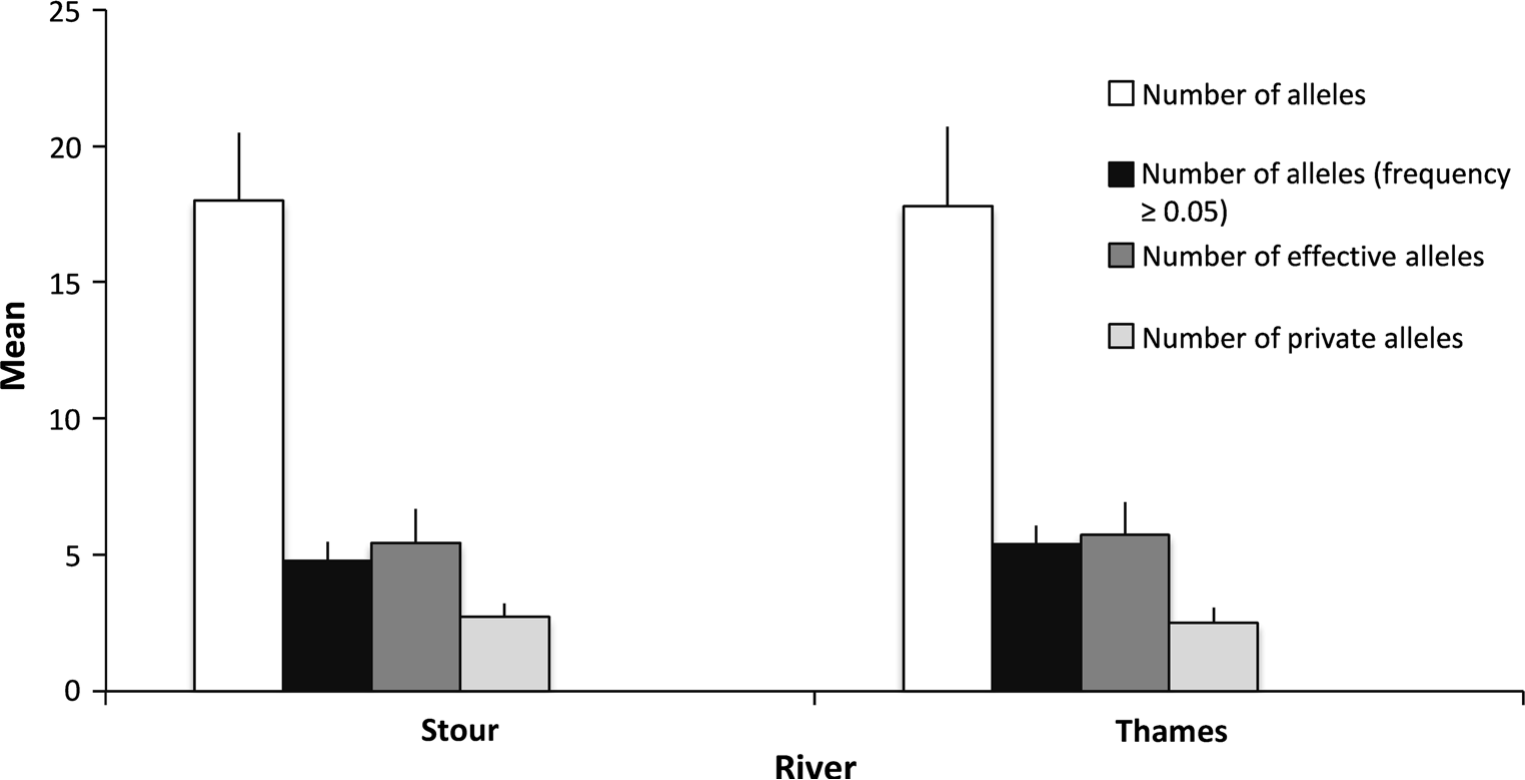
Frequency histograms illustrating allelic variation contained within the Rivers Stour and Thames. *Bars* represent the *upper* limit of the standard error. Student’s *t*-test indicated no statistical difference for any category (*p* > 0.05). The effective number of alleles is the reciprocal of the level of homozygosity across loci, a metric that converges on the actual number of alleles if all alleles are equally frequent (Kimura and Crow 1964). The number of private alleles refers to the number of alleles that are found only in either river

Overall levels of microsatellite diversity were similar for both river populations (Fig. 2) and are consonant with estimates observed in this species in the UK, exhibiting significant overlap between mean and variances in heterozygosity and allelic richness across all surveyed Thames sub-populations (Hamilton et al. 2014). Concordance of diversities is also to be found in European roach (Demandt 2010) and in European coarse fish generally (e.g., Dehais et al. 2010). Each microsatellite locus shows a similar statistical distribution of allele frequencies in either river (Fig S1). However, mean values across the 9 and 13 sub-populations of the Stour and Thames (Table 1), respectively, were significantly different for A_r_ (Mann–Whitney’s U = 114, *p* < 0.001) and H_o_ (Mann–Whitney’s U = 94, *p* = 0.0194), but not for H_e_ (Mann–Whitney’s U = 75, *p* = 0.285) or the mean number of alleles (Mann–Whitney’s U = 39, *p* = 0.182). Of all the diversity metrics, only the mean number of alleles in the Thames increased with downstream distance (R^2^ = 0.450, *p* = 0.012). Similar findings were reported in other cyprinid species that inhabit similarly differently modified river habitats (e.g., chub (*Squalius cephalus*) and dace (*Leucisucus leuciscus*) where demographic instability negatively impacted levels of A_r_ and H_O_ relative to those observed in non-fragmented habitats (Blanchet et al. 2010)).

### Population sub-structuring

**Fig. 3:**
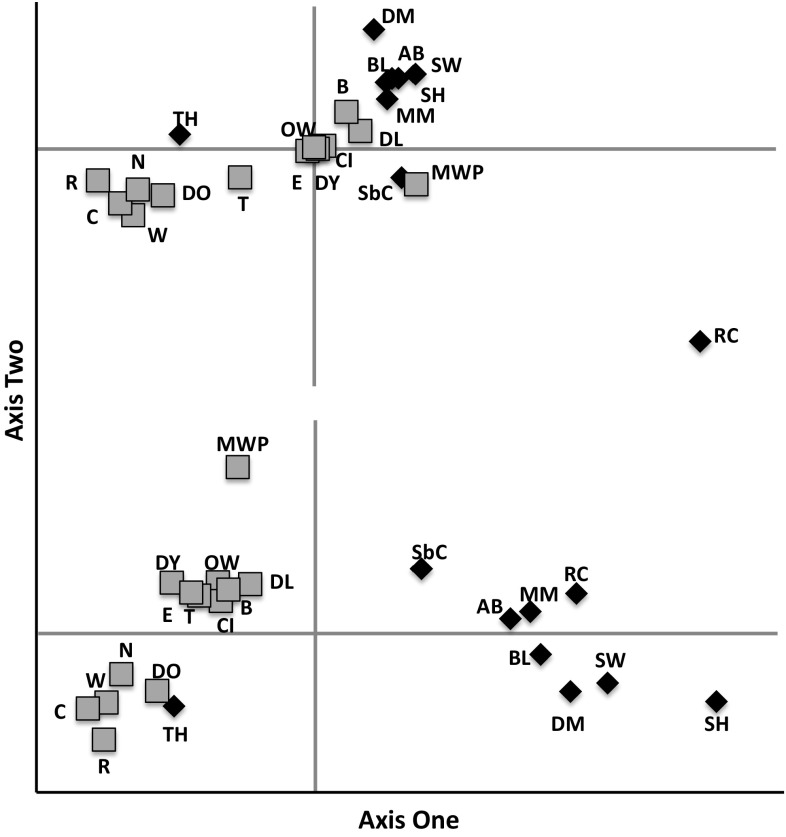
Principal component analysis (PCA) of genetic variation distributed among sub-populations in the Rivers Thames (*grey squares*) and Stour (*black diamonds*). The *upper panel* shows the variance in differentiation based upon allele frequencies (*F*_ST_) that can be apportioned between the first two axes of variation, whereas the *lower panel* displays the principal axes of variation observed among sub-populations based on genotype frequencies (GCD)

Pairwise genetic differentiation was found to be significant in a majority of comparisons in both the Thames (Table 3) and the Stour (Table 4). Of the 78 comparisons in the Thames, G-tests found 67 to be significantly differentiated (86 %). In the Stour, 35 of a possible 36 pairwise comparisons were significant differentiated (97 %). Similarly, the number of significantly differentiated pairwise comparisons computed from *F*_ST_ was lower in the Thames than in the Stour: 72 % and 78 %, respectively. Global *F*_ST_ was found to be significant within both the Thames (0.032, 95 % CI 0.008-0.060, *p* < 0.05) and the Stour (0.039, 95 % CI 0.016–0.043, *p* < 0.05), respectively. Overall mean *F*_ST_ across all sub-populations was 0.036 (95 % CI 0.020–0.062) similar to those described by Hamilton et al. (*F*_ST_ = 0.028) for microsatellite loci and identical to those reported by Hänfling et al. (2004) for allozymes. Although pairwise *F*_ST_ are low, they are statistically non-negligible and imply tangible encumbrances to gene flow (Wright 1978; Balloux and Lugon-Moulin 2002). Each river contained one sub-population that was significantly more divergent than the others: MWP, an area just upstream of a recently stocked stretch of the Thames (mean *F*_ST_ = 0.081) and the headwater population of TH in the Stour (mean *F*_ST_ = 0.085). These sub-populations can be visualised by PCA analysis (Fig. 3; and see Fig S2). The first two axes explain the majority of variance in the system (*F*_ST_: 50.6 %; co-dominant genotypic distance (CGD): 76.7 %). The genotypic distances better delineated the Stour and Thames sub-populations from one another, with both rivers forming cohesive aggregates with the exception of TH. Additionally, both plots suggest MWP, and another upstream Stour site-RC-are distinct. Hierarchical structuring across all sub-populations from both rivers is low but significant, although most of the apportioning of genetic variance was found to be among and within individuals (*F*_IS_ = 0.144, 14 % of the variation, *p* = 0.001; *F*_IT_ = 0.186, 81 % of the variation, *p* = 0.001). The degree of genetic diversity apportioned between sub-populations within rivers and between rivers accounts for 5 % of the entire total variation (*F*_SC_ = 0.033, 3 % of the variation, *p* = 0.001; *F*_CT_ = 0.017, 2 % of the variation) of a similar scale to that previously observed for UK roach (Hamilton et al. 2014).

**Table 3:** Pairwise estimates of genetic differentiation between sub-populations in the River Thames based on allele frequencies at ten microsatellite loci

	Upstream												
	MWP	DL	OW	CI	T	W	DO	DY	CI	E	N	B	R
MWP		HS	HS	HS	HS	HS	HS	HS	HS	HS	HS	HS	HS
DL	*0.062*		HS	0.009	HS	HS	HS	HS	HS	0.156	HS	HS	HS
OW	*0.062*	0.011		HS	HS	HS	HS	HS	HS	0.497	HS	0.005	HS
CI	*0.071*	0.013	0.005		HS	HS	HS	HS	HS	0.017	HS	HS	HS
T	*0.070*	*0.021*	0.006	*0.013*		HS	HS	HS	HS	0.003	HS	HS	HS
W	*0.103*	*0.045*	0.029	*0.037*	*0.024*		0.001	HS	HS	HS	0.077	HS	0.004
DO	*0.097*	*0.036*	*0.028*	*0.037*	*0.018*	0.005		HS	HS	HS	HS	HS	HS
DY	*0.061*	*0.023*	*0.031*	*0.038*	*0.033*	*0.057*	*0.044*		HS	HS	HS	HS	HS
CI	*0.109*	*0.053*	*0.041*	*0.044*	*0.021*	0.014	0.011	*0.066*		HS	HS	HS	HS
E	*0.060*	0.004	0.001	0.005	0.005	*0.040*	*0.028*	*0.024*	*0.039*		HS	0.252	HS
N	*0.092*	*0.041*	*0.025*	*0.027*	*0.019*	0.006	0.010	*0.056*	*0.019*	*0.025*		HS	0.307
B	*0.084*	0.009	0.004	0.011	0.012	*0.045*	*0.031*	*0.036*	*0.038*	0.001	*0.036*		HS
R	*0.098*	*0.046*	*0.037*	*0.040*	*0.028*	0.009	0.005	*0.048*	*0.015*	*0.033*	−0.003	*0.044*	

The figures above the diagonal show the significance of differentiation based on the G-test adopted in Genepop. HS indicates a highly significant level of differentiation (<0.001). Below the diagonal each cell indicates the level of differentiation inferred from Cockerham and Weir’s estimator of *F*_ST_, θ. Statistically significant values are indicated by *italics*. All significant values adopt a Bonferroni-corrected alpha-level of 0.05

**Table 4:** Pairwise estimates of genetic differentiation between sub-populations in the River Stour based on allele frequencies at ten microsatellite loci

	Upstream								
	BL	DM	SW	AB	SH	MM	RC	SbC	TH
BL		HS	HS	HS	0.020	HS	HS	HS	HS
DM	*0.024*		HS	HS	HS	HS	HS	HS	HS
SW	0.007	*0.014*		HS	HS	HS	HS	HS	HS
AB	*0.024*	0.022	*0.013*		HS	HS	HS	HS	HS
SH	0.000	*0.026*	0.011	*0.020*		HS	HS	HS	HS
MM	0.009	*0.026*	0.011	*0.018*	0.010		HS	HS	HS
RC	*0.056*	*0.092*	*0.038*	*0.035*	*0.063*	*0.047*		HS	HS
SbC	*0.045*	*0.074*	*0.030*	*0.038*	*0.056*	*0.037*	0.018		HS
TH	*0.085*	*0.086*	*0.084*	*0.060*	*0.097*	*0.080*	*0.101*	*0.086*	

The figures above the diagonal show the significance of differentiation based on the G-test adopted in Genepop. HS indicates a highly significant level of differentiation (<0.001). Below the diagonal each cell indicates the level of differentiation inferred from Cockerham & Weir’s estimator of *F*_ST_, θ. Statistically significant values are indicated by *italics*. All significant values adopt a Bonferroni-corrected alpha-level (0.05)

### Isolation by distance and population connectivity

**Fig. 4:**
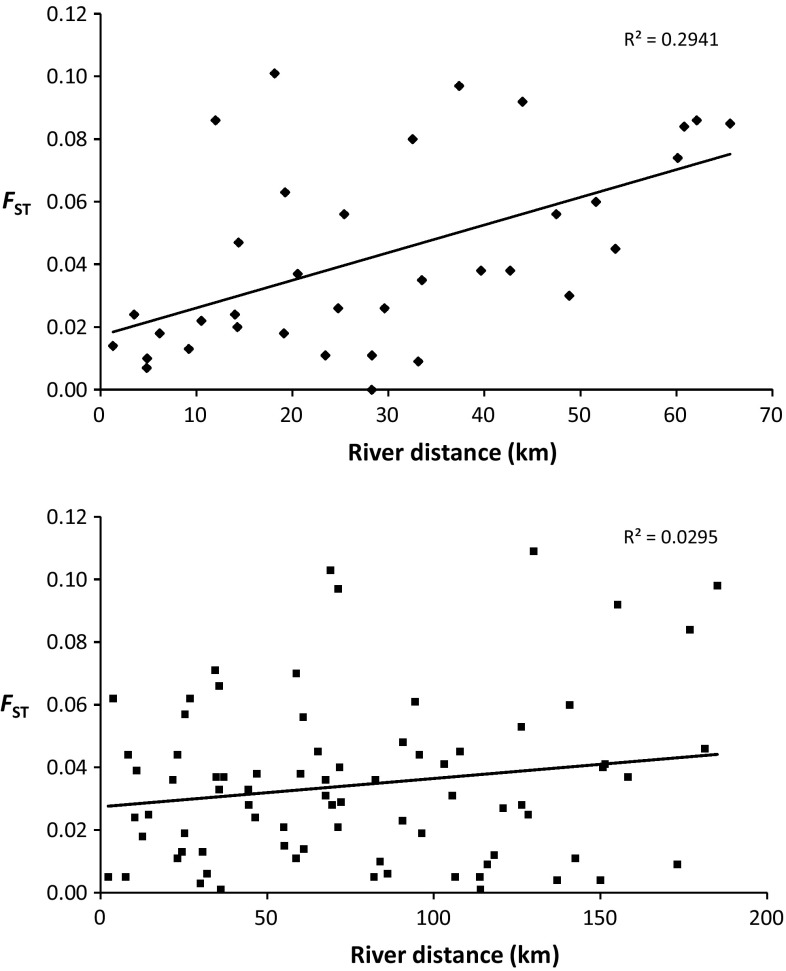
Scatter plots illustrating the pairwise relationship between genetic distance (*F*_ST_) and geographic distances (km) between sub-populations in both the Rivers Stour (*top*) and Thames (*bottom*). *Lines* of best fit and R^2^ values are shown

**Fig. 5:**
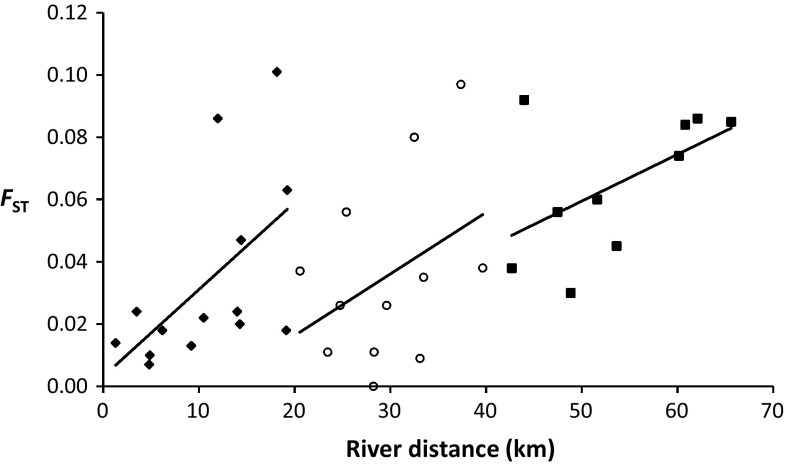
Scatter graph from Fig. 4 illustrating Type I IBD in the Stour through increasing 20–25 km segments (indicated by *solid diamonds*, *open circles* and *solid squares*, respectively) highlighting a lack of plateauing at increasing spatial scales

Both rivers exhibited a positive correlation between genetic structuring and distance (Fig. 4), although the Thames was not significant (Thames: R^2^ = 0.034, *p* = 0.087; Stour: R^2^ = 0.340, *p* = 0.001). Because divergent outlier populations may overestimate the degree to which IBD is observed (Schwartz and McKelvey 2009), the analysis was repeated without TH in the Stour. The signal of IBD remained strong in the Stour (R^2^ = 0.403, *p* = 0.006). Following Hänfling and Weetman (2006), regressing *F*_ST_ and distance at smaller scales allowed a closer examination of the relationship between *F*_ST_ and distance (Fig. 5). By focussing on IBD in two 20 and a final 25 km distance windows, we find that there is no tendency for IBD to plateau at increasing spatial scales, suggesting that IBD is maintained through regional equilibrium processes (i.e. representative of a Type I relationship, sensu Hutchinson and Templeton (1999), between genetic and geographic distances).

Partial Mantels tests revealed only a single, consistent predictive variable of genetic divergence among populations: the number of tributaries (Table 5). Unsampled ‘ghost’ populations (Beerli 2004) may contribute gene flow to genotyped sub-populations, although their effects are expected to be low when *F*_ST_s are significant (Strasburg and Rieseberg 2009). Tributaries likely provide spawning areas which roach may utilise in addition to those in the main stem thereby contributing to the positive correlation with *F*_ST_. In both rivers, the number of gauging weirs was significantly positively correlated with *F*_ST_ prior to controlling for distance (*p* < 0.01; Fig S3), remaining borderline significant in the Thames after accounting for geography (r = 0.216, *p* = 0.057). However, the Partial Mantel test indicated a reversal of correlation in the Stour when a third variable-distance-was controlled for—an example of the so-called Simpson’s Paradox (Tu et al. 2008; Fig S3). This result is indicative of multicollinearity of distance variables in the Stour, whereby correlations between variables leads to negative correlations in one or more of the estimated coefficients. As far as we are aware, this is the first reporting of Simpson’s Paradox using environmental distance data in Partial Mantel tests. They are clearly egregious for interpretation of environmental and genetic distance relationships.

**Table 5:** Table of Partial Mantel tests correlations (r) and *p* values showing the correlation between river factors and *F*_ST_ when geographic distance is accounted for

River	Factor	*F*_ST_	
		*r*	*p*
Thames	Oestrone (ng L^−1^)	−0.123	0.255
	Flow (m^3 s−1^)	−0.112	0.242
	No. weirs	0.216	0.057
	No. tributaries	0.480	0.030
	Distance (km)	0.174	0.099
Stour	Oestrone (ng L^−1^)	0.010	0.397
	Flow (m^3 s−1^)	−0.384	0.021
	No. weirs	−0.377	0.093
	No. tributaries	0.599	0.002
	Distance (km)	0.543	0.002

The simple Mantel correlation between *F*_ST_ and distance is also shown

**Fig. 6:**
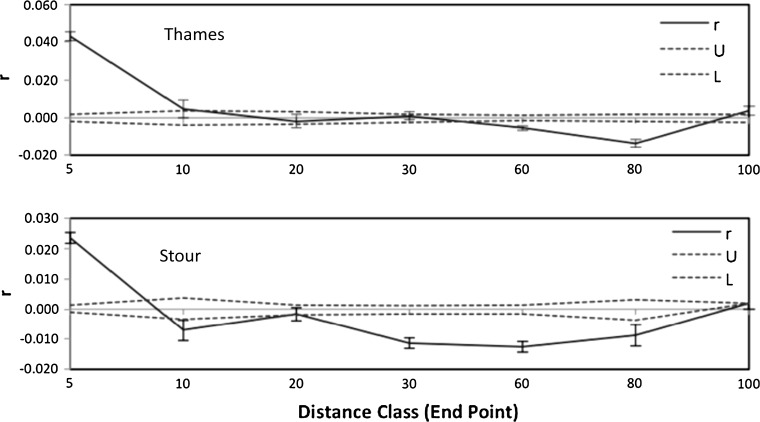
Genetic autocorrelation plots. The *upper* and *lower* *panels* show the decreasing correlation (r) between genetic similarity and distance class for pairwise comparisons of individual roach sampled within the Thames and Stour, respectively. The dotted lines represent the 95 % confidence interval (CI) limits around the null hypothesis of no difference. The *error bars* indicate 95 % CI for each point estimate of genetic similarity

The distance at which genetic similarity between individuals became statistically independent was assessed by application of a genetic autocorrelation analysis (Fig. 6). The autocorrelation coefficient was shown to be higher than expected by chance alone within the first two distance classes of 5 and 10 km within the Thames (*p* = 0.001 and 0.010, respectively), and in the 5 km class (*p* = 0.010) within the Stour. Correspondingly, the point at which the plot of *r* intercepts with distance class is approximately twice as distant in the Thames (17.10 km) than it is in the Stour (8.85 km). These data suggest that the distance above which gene flow no longer effectively counteracts allelic frequency divergence caused by genetic drift is greater in the Thames.

Contemporaneous rates of migration are high (Tables S3 and S4). Both rivers experience significant gene flow (>0.7, i.e. the probability that a sampled individual in a given sub-population is a recent immigrant is 70 %). High connectivity complicates interpretation on a system-wide scale when global *F*_ST_ is low (<0.05) (Meirmans 2014) despite convergence of MCMC chains and the expectation of spatially-delimited gene flow in our data. Nonconvergence precluded an analysis of long-term gene flow using N-MIGRATE (Beerli and Felsenstein 2001). Therefore, these results must be applied cautiously. Nonetheless, the potential for uncovering significant sinks and sources (i.e. potential spawning and recruitment areas) of migrants remains. MWP in the Thames exhibited relatively low rates of immigration (≤0.1), deriving from the two adjacent sub-populations of DL and OW. Similarly, relatively low levels of immigration were exhibited by OW (0.1430) and DO (0.1490), collectively inferred to be the source of most migrants within the Thames. In the Stour, the most downstream and the most upstream sub-populations at BL and TH, respectively, receive fewer migrants from all other sub-populations (0.0650 and 0.1350, respectively). For all adjacent sub-populations located between DM and MM, the highest proportion of immigrants all derived from BL (0.2820–0.2770). However, the next two adjacent sub-populations—RC and SbC—received the most migrants from SW (0.2430 and 0.2720, respectively).

### Sub-population bottlenecks and N_e_

**Fig. 7:**
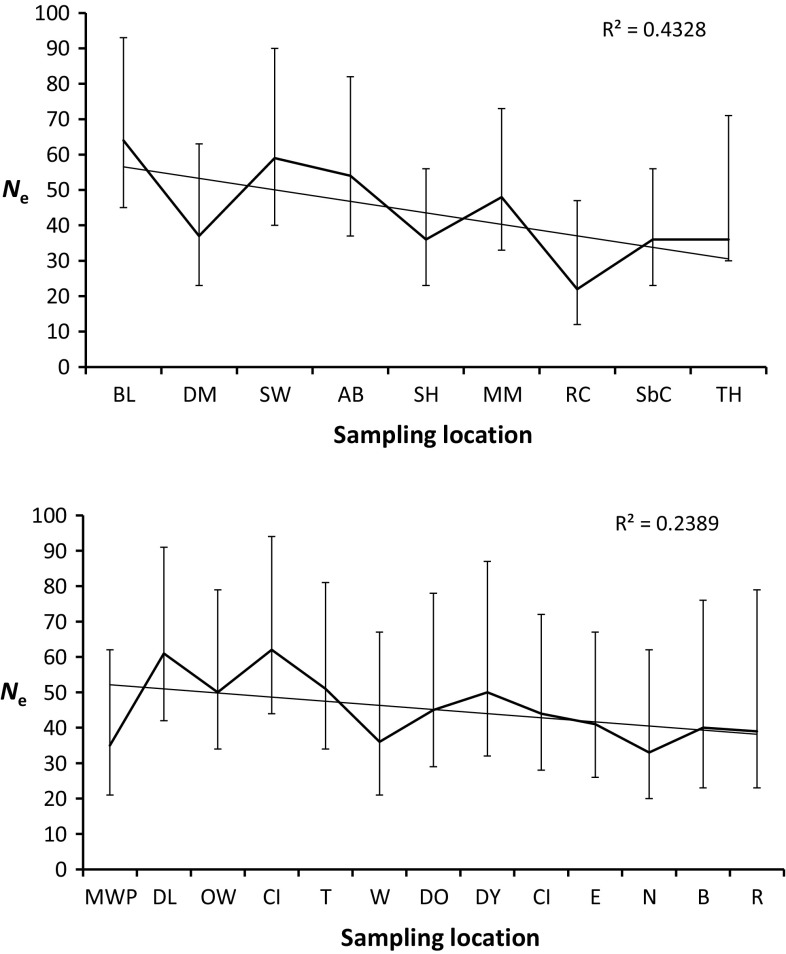
Line-graphs depicting a decrease in the effective sizes of sub-populations (*N*_e_) with upstream location (95 % confidence intervals shown as *vertical bars*) for the nine sub-populations in the Stour (*top*) and the 13 sub-populations in the Thames. However, note the low *N*_e_ of the MWP population, which has recently been subject to stocking by exogenous fish

*N*_e_ was estimated for each of 22 sub-populations (Fig. 7). In the Thames, *N*_e_ ranged from 35 (MWP: 95 % CI 21–62) to 62 (CI 95 % CI 44–94). Mean Thames *N*_e_ was 45 (95 % CI 29–77). In the Stour, *N*_e_ ranged from 22 (RC: 95 % CI 12-47) to 64 (BL: 95 % CI 45–93). Mean Stour *N*_*e*_ was 43 (95 % CI 30–70). Mean *N*_e_ across sub-populations between the two rivers was not significantly different, providing no support to the prediction that mean *N*_e_ in the Stour would be lower. For both the Thames and the Stour, there exists a negative linear correlation between distance upstream and the *N*_e_ of the sub-population (Fig. 7; Thames: R^2^ = 0.239, *p* = 0.054; Stour: R^2^ = 0.432, *p* = 0.019) indicating a significant influence of genetic drift on the standing variation of upstream sub-populations. After correcting for multiple comparisons, the BOTTLENECK analysis inferred no demographic contraction in the Thames nor in the Stour, although MM and TH were borderline significant in the latter (*p* = 0.007 and 0.009, respectively; corrected alpha = 0.005).

## Discussion

We investigated patterns of genetic structuring and gene flow in a very common sport fish with demonstrably high within-population genetic variability and low levels of intraspecific differentiation (Bouvet et al. 1991, 1995; Baranyi et al. 1997; Wolter 1999; Hänfling et al. 2004; Demandt 2010; Hamilton et al. 2014). Whilst a strong signal of IBD was only detected in the Stour, both rivers possessed idiosyncratic patterns of divergence.

### Drivers of genetic differentiation and population connectivity

Microsatellite variability observed in this study falls within the range of that observed across European populations (Wolter 1999; Hänfling et al. 2004). Despite significant range contraction during the Pleistocene, northerly distributed coarse fishes may retain large evolutionary *N*_e_ maintaining high genetic variability (Larmuseau et al. 2009). Similar levels of allelic diversity in the Thames and Stour (Fig. 2, Fig S1) are likely the result of contemporaneous processes that maintain high levels of genetic diversity across both rivers. However, the distribution of allelic variation among sub-populations does differ between the two rivers due to the physical and/or biotic properties of the two systems. Mean A_r_ and H_O_ was significantly lower in the Stour, whereas mean *F*_ST_ was higher, consistent with a scenario of localised demographic instability and population fragmentation (Blanchet et al. 2010). This scenario is supported by the observation that four sub-populations in the Stour showed signs of a recent population contraction, albeit prior to Bonferroni correction. Population contractions tend to ratchet population-level inbreeding increasing the proportion of homozygotes relative to heterozygotes, explaining the higher incidence of Hardy–Weinberg disequilibrium within the Stour, and the aggregation of sub-populations according to genotypic frequency, rather than allelic frequency (Fig. 3), although estimates of mean *N*_e_ between the two rivers were unaffected.

Overall, genetic structuring among sub-populations was low, consistent with patterns of neutral genetic variation observed across coarse fishes (e.g., Dehais et al. 2010; Blanchet et al. 2010). However, MWP and TH are significantly divergent to warrant individual attention. MWP’s genetic signature is likely representative of exogenous allelic variation introduced to the immediate downstream vicinity thorough the stocking of roach at Teddington (51.470042 latitude, −0.321241 longitude) and London Apprentice (51.432433 latitude, −0.326071 longitude) (Nigel Hewlett pers comm)). The low *N*_e_ of MWP is consistent with low levels of genetic diversity associated with the population-level inbreeding effects of founder populations. The genetic constitution of the introduced roach is unknown, as is the exact location of the lake from which the roach were taken, so explicit testing of this hypothesis is difficult. By contrast, both anthropogenic and natural processes may explain the divergence of TH and, to a lesser extent, RC. Both these sub-populations are located upstream of a significant water extraction point at Wixoe (Fig. 1), periodically affecting water levels and curtailing connectivity from below. Headwater populations, subject to asymmetric immigration, will be further affected by low flow acting in concert to drive divergence further. Although these areas are vulnerable to bottlenecking, the same signal is compatible with a scenario of re-colonisation post-extirpation (Hänfling and Weetman 2006).

IBD is expected in riverine freshwater fishes but it is not universally found in all river populations (Hänfling et al. 2004; Dehais et al. 2010; Hamilton et al. 2014), corroborating our findings. The strength of IBD may also vary over generations, complicating biological interpretation of snapshot estimates (Junge et al. 2011). Nevertheless, a strong signal of IBD was observed in the Stour. Genetic drift acting during spatially independent bottleneck or founding events should manifest in a Type IV IBD pattern, but this was not observed in the Stour. Instead high gene flow maintains IBD at all scales (Fig. 5), which is borne out by high levels of migration into most populations from BL and from SW (Table S4). Regression analysis of the residuals derived from Mantel tests indicate that removal of TH strengthens the Type I pattern by increasing scatter (r = 0.539, *p* = 0.008), consistent with non-equilibrium conditions. Further confidence in this conclusion is provided by TH receiving fewer migrants than elsewhere, with the exception of BL. The direction of net migration in both rivers was biased in the upstream direction, consonant with known migratory behaviour of roach (Lucas and Baras 2001; but see Champion and Swain 1974). Diversity may be maintained in non-headwater upstream reaches by active migration from downstream sources providing propagules for sink regions, but these conclusions must be caveated against the limitations of Bayesian inference when genetic structuring is low. Further, confidence in the occurrence of non-equilibrium conditions derived from genetic data are tempered by the fact that asymmetrical gene flow may result in spurious BOTTLENECK results (Paz-Vinas et al. 2013), possibly explaining a pre-Bonferroni corrected inferred bottleneck at BL, the single most important source of migrants in the Stour.

The number of weirs seems to have some influence upon differentiation in the Thames, but the suitability of Mantel tests to disentangle the effects of potentially confounding variables is questionable. Many weirs are only passable during high flows, severely limiting the opportunity for upstream migration whilst allowing the passive drift of juveniles during these periods. Biotic factors such as size may also determine differential population connectivity between rivers. Sampled roach in the Thames were larger than those from the Stour (heteroscedastic Student’s *t* test, *p* < 0.01) possibly enabling greater long-distance dispersal (Radinger and Wolter 2013). Furthermore, size is negatively correlated with dispersal timing in Norwegian roach (Vøllestad and L’Abée-Lund 1987). A combination of high water levels from winter run-off and larger size may enable some individuals to migrate earlier and further during the spawning season (April-June) as they would be better equipped to bypass shallower obstacles in the river channel.

### Implications for management

Patterns of genetic differentiation and gene flow were different in both rivers, therefore each population should be considered as independent management units. Type I IBD and long-distance dispersal ensures the ability for nearby areas to replenish neighbouring and distant reaches in the event of fish-kills, implying these rivers may be able to re-populate themselves given time. It seems that there are fewer impediments to gene flow within the River Thames, although it seems likely that weirs of varying size play an integral role in promulgating contemporaneous genetic structuring. The construction of fish passes will help mitigate against localised bottlenecking, the deleterious effects of inbreeding and further enable recolonisation of depauperate areas. The abstraction of water for agricultural and municipal usage needs to be balanced against the potential damage to upstream sub-populations caused by limiting inbound migrants. More positively, significant sources of immigrants were inferred in both rivers, providing management with information for the approximate location of spawning areas for cyprinids in general as they often share similar phytolithophilic substrate (Mann 1996). Furthermore, it seems likely that a pervasive signal from a recent stocking event was evident at MWP. Although this site is close to the tidal reach of the Thames, we reject the possibility that these populations are divergent due to ecological selection for salinity tolerance as this is upstream of the beginning of the halocline. 6 years is likely too short a time to determine long-term impacts of stocking, but MWP neither received nor sourced many effective migrants at the time of surveying, although telemetric data suggest that stocked roach disperse up and downstream from sites of introduction (Bollard et al. 2009). A potential lack of assimilation through outbreeding depression (Templeton 1986) or within-population breeding (through asynchrony of spawning migrations or by assortative mating (Almodóvar et al. 2006)) would prove wasteful for the limited resources available for managing complex riverine ecosystems.

## Conclusions

 This study contributes further data on the spatial variability of IBD among freshwater fishes. Although significant pairwise genetic differentiation was observed in both rivers, we were unable to infer significant anthropogenic impediments to gene flow, although we suspect analytical autocorrelation among distance data to obscure a significant result. Clearly, further refinement of the statistical robustness of such tests and the addition of more data is necessary for the reliable inference of the environmental drivers of contemporary gene flow. The two most divergent sub-populations likely had different underlying causation: retention of ancestral allele frequencies in a stocked population (MWP); and a combination of headwater demography and periodic, anthropogenic low-flow rates (TH). Although just two rivers were compared, the results suggest that the idiosyncratic demographic, historical and natural features of individual riverine habitats will result in unique patterns of both IBD and genetic divergence within and between populations and species. A greater knowledge of population connectivity across species and lentic water bodies will greatly improve the ability to restore and maintain wild stocks of aquatic biodiversity.

## Electronic supplementary material

Below is the link to the electronic supplementary material.

Supplementary material 1 (DOCX 1501 kb)
