# Supplementary material for: Isolation by distance and non-identical patterns of gene flow within two river populations of the freshwater fish Rutilus rutilus (L. 1758)
Source: Conserv Genet. 2016 Mar 10;17(4):861–74. doi: 10.1007/s10592-016-0828-3 (PMC7175732; doi:10.1007/s10592-016-0828-3)
Supplement: Supplementary file 1 — Supplementary material 1 (DOCX 1501 kb) [file 10592_2016_828_MOESM1_ESM.docx]

**Title:** Isolation by distance and non-identical patterns of gene flow within two river populations of the freshwater fish *Rutilus rutilus* (L. 1758).

**Journal:** Conservation Genetics

**Authors:** S Crookes & P Shaw

**Affiliation:** Royal Holloway, University of London, Egham, London**.**

**Corresponding author:** scrookes@uwindsor.ca

|  | **Sampling location** | | | | | | | | | | | | |
| --- | --- | --- | --- | --- | --- | --- | --- | --- | --- | --- | --- | --- | --- |
| **Locus** | **MWP** | **DL** | **OW** | **CI** | **T** | **W** | **DO** | **DY** | **C** | **Y** | **N** | **B** | **R** |
| **Rru3** |  |  |  |  |  |  |  |  |  |  |  |  |  |
| N_A_ | 5.000 | 5.000 | 4.000 | 7.000 | 5.000 | 5.000 | 4.000 | 5.000 | 4.000 | 5.000 | 7.000 | 5.000 | 5.000 |
| A_R_ | 4.892 | 4.693 | 3.984 | 5.534 | 4.028 | 5.000 | 4.000 | 4.508 | 3.987 | 4.489 | 6.315 | 4.721 | 4.989 |
| H_e_ | 0.596 | 0.576 | 0.567 | 0.644 | 0.492 | 0.652 | 0.661 | 0.578 | 0.587 | 0.528 | 0.665 | 0.471 | 0.661 |
| H_o_ | 0.500 | 0.290 | 0.479 | 0.351 | 0.451 | 0.588 | 0.476 | 0.333 | 0.500 | 0.364 | 0.500 | 0.391 | 0.636 |
| *F*_IS_ | 0.163 | 0.498** | 0.156 | 0.458** | 0.084 | 0.101 | 0.284 | 0.427 | 0.150** | 0.315** | 0.252 | 0.173 | 0.038 |
| **Lid1** |  |  |  |  |  |  |  |  |  |  |  |  |  |
| N_A_ | 8.000 | 10.000 | 10.000 | 8.000 | 9.000 | 10.000 | 7.000 | 9.000 | 9.000 | 10.000 | 6.000 | 6.000 | 6.000 |
| A_R_ | 6.450 | 7.881 | 7.910 | 6.174 | 6.156 | 8.888 | 5.810 | 8.206 | 6.321 | 8.439 | 5.857 | 5.192 | 5.429 |
| H_e_ | 0.702 | 0.813 | 0.743 | 0.657 | 0.676 | 0.776 | 0.729 | 0.811 | 0.649 | 0.741 | 0.644 | 0.655 | 0.668 |
| H_o_ | 0.563 | 0.609 | 0.583 | 0.541 | 0.632 | 0.652 | 0.613 | 0.484 | 0.750 | 0.750 | 0.652 | 0.731 | 0.619 |
| *F*_IS_ | 0.201 | 0.252** | 0.216 | 0.177 | 0.067 | 0.162 | 0.161 | 0.407** | -0.157 | -0.012 | -0.012 | -0.119 | 0.075 |
| **CypG3** |  |  |  |  |  |  |  |  |  |  |  |  |  |
| N_A_ | 17.000 | 18.000 | 16.000 | 20.000 | 22.000 | 15.000 | 15.000 | 13.000 | 18.000 | 14.000 | 15.000 | 15.000 | 13.000 |
| A_R_ | 12.426 | 11.097 | 11.721 | 12.486 | 13.114 | 13.342 | 11.078 | 9.767 | 12.662 | 10.756 | 12.841 | 11.985 | 11.287 |
| H_e_ | 0.755 | 0.746 | 0.811 | 0.870 | 0.798 | 0.818 | 0.706 | 0.700 | 0.785 | 0.720 | 0.808 | 0.739 | 0.697 |
| H_o_ | 0.679 | 0.600 | 0.667 | 0.724 | 0.772 | 0.682 | 0.656 | 0.645 | 0.692 | 0.727 | 0.955 | 0.654 | 0.545 |
| *F*_IS_ | 0.103 | 0.197 | 0.180 | 0.169 | 0.033 | 0.170 | 0.072 | 0.079 | 0.119 | -0.011 | -0.187 | 0.117 | 0.221 |
| **CypG48** |  |  |  |  |  |  |  |  |  |  |  |  |  |
| N_A_ | 15.000 | 16.000 | 12.000 | 14.000 | 14.000 | 11.000 | 13.000 | 13.000 | 11.000 | 12.000 | 13.000 | NA | 11.000 |
| A_R_ | 12.396 | 11.138 | 10.246 | 10.827 | 10.509 | 9.631 | 11.209 | 11.041 | 9.400 | 10.017 | 11.880 | NA | 10.039 |
| H_e_ | 0.912 | 0.889 | 0.864 | 0.884 | 0.885 | 0.850 | 0.909 | 0.896 | 0.840 | 0.889 | 0.914 | NA | 0.888 |
| H_o_ | 0.939 | 0.922 | 0.700 | 0.857 | 0.759 | 0.739 | 0.844 | 0.879 | 0.816 | 0.625 | 0.769 | NA | 0.900 |
| *F*_IS_ | -0.030 | -0.037 | 0.193 | 0.031 | 0.144 | 0.133 | 0.073 | 0.019 | 0.029 | 0.300** | 0.161 | NA | -0.013 |
| **Ca1** |  |  |  |  |  |  |  |  |  |  |  |  |  |
| N_A_ | 8.000 | 12.000 | 13.000 | 14.000 | 10.000 | 9.000 | 6.000 | 8.000 | 9.000 | 10.000 | 9.000 | 10.000 | 7.000 |
| A_R_ | 7.453 | 8.625 | 8.867 | 9.407 | 7.200 | 7.829 | 5.371 | 6.820 | 7.266 | 8.147 | 8.066 | 9.034 | 6.554 |
| H_e_ | 0.796 | 0.822 | 0.792 | 0.839 | 0.783 | 0.743 | 0.711 | 0.796 | 0.764 | 0.798 | 0.813 | 0.845 | 0.753 |
| H_o_ | 0.760 | 0.683 | 0.818 | 0.727 | 0.698 | 0.652 | 0.600 | 0.719 | 0.882 | 0.645 | 0.440 | 0.875 | 0.619 |
| *F*_IS_ | 0.046 | 0.170 | -0.034 | 0.134 | 0.110 | 0.125 | 0.158 | 0.098 | -0.158 | 0.194 | 0.464** | -0.036 | 0.181 |
| **Ca3** |  |  |  |  |  |  |  |  |  |  |  |  |  |
| N_A_ | 16.000 | 17.000 | 17.000 | 19.000 | 22.000 | 15.000 | 18.000 | 16.000 | 16.000 | 19.000 | 16.000 | 18.000 | 16.000 |
| A_R_ | 13.909 | 14.391 | 13.486 | 14.269 | 16.059 | 12.980 | 15.167 | 13.483 | 13.402 | 15.956 | 14.225 | 15.168 | 14.744 |
| H_e_ | 0.929 | 0.936 | 0.928 | 0.931 | 0.944 | 0.903 | 0.940 | 0.911 | 0.926 | 0.941 | 0.925 | 0.934 | 0.931 |
| H_o_ | 0.839 | 0.931 | 0.870 | 0.917 | 0.947 | 0.870 | 0.700 | 0.724 | 0.730 | 0.750 | 0.833 | 0.800 | 0.667 |
| *F*_IS_ | 0.099 | 0.005 | 0.064 | 0.015 | -0.003 | 0.038 | 0.258 | 0.208 | 0.215 | 0.206** | 0.101 | 0.146 | 0.289 |
| **Ca12** |  |  |  |  |  |  |  |  |  |  |  |  |  |
| N_A_ | 17.000 | 21.000 | 23.000 | 23.000 | 19.000 | 16.000 | 15.000 | 15.000 | 17.000 | 13.000 | 13.000 | 16.000 | 15.000 |
| A_R_ | 12.850 | 13.246 | 13.994 | 13.378 | 13.081 | 14.056 | 11.845 | 11.255 | 13.076 | 10.327 | 10.456 | 13.010 | 13.161 |
| H_e_ | 0.869 | 0.805 | 0.854 | 0.830 | 0.854 | 0.865 | 0.829 | 0.785 | 0.864 | 0.732 | 0.676 | 0.793 | 0.784 |
| H_o_ | 0.897 | 0.800 | 0.857 | 0.726 | 0.817 | 0.818 | 0.727 | 0.636 | 0.794 | 0.697 | 0.680 | 0.720 | 0.682 |
| *F*_IS_ | -0.032 | 0.006 | -0.003 | 0.126 | 0.044 | 0.055 | 0.124 | 0.192** | 0.082 | 0.048 | -0.006 | 0.093 | 0.133 |
| **Lc27** |  |  |  |  |  |  |  |  |  |  |  |  |  |
| N_A_ | 3.000 | 5.000 | 4.000 | 3.000 | 4.000 | 3.000 | 4.000 | 3.000 | 3.000 | 4.000 | 4.000 | 3.000 | 4.000 |
| A_R_ | 2.746 | 3.568 | 3.355 | 2.263 | 2.955 | 2.739 | 3.746 | 2.514 | 2.892 | 3.308 | 3.304 | 2.952 | 3.848 |
| H_e_ | 0.197 | 0.311 | 0.243 | 0.225 | 0.227 | 0.300 | 0.426 | 0.265 | 0.287 | 0.255 | 0.246 | 0.247 | 0.419 |
| H_o_ | 0.152 | 0.271 | 0.245 | 0.222 | 0.200 | 0.261 | 0.455 | 0.242 | 0.275 | 0.219 | 0.269 | 0.231 | 0.350 |
| *F*_IS_ | 0.234 | 0.130 | -0.008 | 0.014 | 0.121 | 0.134 | -0.069 | 0.087 | 0.042 | 0.146 | -0.097 | 0.065 | 0.169 |
| **Lc290** |  |  |  |  |  |  |  |  |  |  |  |  |  |
| N_A_ | 10.000 | 10.000 | 8.000 | 8.000 | 9.000 | 7.000 | 8.000 | 9.000 | 9.000 | 10.000 | 7.000 | 10.000 | 10.000 |
| A_R_ | 8.448 | 7.514 | 6.592 | 6.743 | 7.105 | 6.887 | 7.213 | 7.096 | 8.138 | 8.269 | 6.621 | 9.226 | 9.360 |
| H_e_ | 0.795 | 0.801 | 0.746 | 0.791 | 0.756 | 0.797 | 0.821 | 0.723 | 0.786 | 0.808 | 0.798 | 0.869 | 0.814 |
| H_o_ | 0.636 | 0.677 | 0.694 | 0.730 | 0.754 | 0.667 | 0.594 | 0.485 | 0.553 | 0.710 | 0.680 | 0.630 | 0.800 |
| *F*_IS_ | 0.202 | 0.155 | 0.070 | 0.077 | 0.002 | 0.167 | 0.280 | 0.333 | 0.299 | 0.124 | 0.150 | 0.279 | 0.018 |
| **Lco4** |  |  |  |  |  |  |  |  |  |  |  |  |  |
| N_A_ | 5.000 | 5.000 | 5.000 | 6.000 | 7.000 | 4.000 | 6.000 | 6.000 | 6.000 | 6.000 | 6.000 | 5.000 | 6.000 |
| A_R_ | 4.357 | 4.300 | 4.570 | 4.916 | 6.542 | 3.991 | 5.891 | 5.671 | 5.806 | 4.922 | 5.763 | 4.796 | 5.722 |
| H_e_ | 0.449 | 0.621 | 0.691 | 0.646 | 0.778 | 0.673 | 0.769 | 0.707 | 0.743 | 0.613 | 0.755 | 0.643 | 0.714 |
| H_o_ | 0.364 | 0.656 | 0.646 | 0.508 | 0.714 | 0.500 | 0.710 | 0.548 | 0.600 | 0.531 | 0.500 | 0.520 | 0.773 |
| *F*_IS_ | 0.192 | -0.057 | 0.066 | 0.215 | 0.083 | 0.262 | 0.079 | 0.227 | 0.195 | 0.135 | 0.343 | 0.195 | -0.085 |
| **Mean** |  |  |  |  |  |  |  |  |  |  |  |  |  |
| N_A_ | 10.400 | 11.900 | 11.200 | 12.200 | 12.100 | 9.500 | 9.600 | 9.700 | 10.200 | 10.300 | 9.600 | 9.778 | 9.300 |
| A_R_ | 8.592 | 8.645 | 8.473 | 8.600 | 8.675 | 8.534 | 8.133 | 8.036 | 8.223 | 8.463 | 8.533 | 8.454 | 8.475 |
| H_e_ | 0.700 | 0.732 | 0.724 | 0.732 | 0.719 | 0.738 | 0.750 | 0.717 | 0.723 | 0.703 | 0.724 | 0.688 | 0.733 |
| H_o_ | 0.633 | 0.644 | 0.656 | 0.630 | 0.674 | 0.643 | 0.637 | 0.570 | 0.659 | 0.602 | 0.628 | 0.617 | 0.659 |
| *F*_IS_ | 0.098 | 0.121** | 0.095** | 0.140** | 0.063 | 0.131 | 0.152** | 0.208** | 0.089** | 0.145** | 0.136** | 0.106 | 0.103** |

Table S1. Individual locus-by-location estimates of genetic variation observed within the River Thames. N_A_ = the number of unique alleles, A_r_ = allelic richness, H_e_ = expected heterozygosity, H_o_ = observed heterozygosity, *F*_IS_ = population-level inbreeding coefficient (underlined values indicate a significant deviation of allele frequencies from those expected under a null model of Hardy-Weinberg equilibrium; values with * are significant before Bonferroni correction).

|  | **Sampling location** | | | | | | | | |
| --- | --- | --- | --- | --- | --- | --- | --- | --- | --- |
| **Locus** | **BL** | **DM** | **SW** | **AB** | **SH** | **MM** | **RC** | **SbC** | **T** |
| **Rru3** |  |  |  |  |  |  |  |  |  |
| N_A_ | 6.000 | 6.000 | 7.000 | 13.000 | NA | 8.000 | 7.000 | 5.000 | 6.000 |
| A_R_ | 4.659 | 5.227 | 3.998 | 7.906 | NA | 4.933 | 6.402 | 4.320 | 5.206 |
| H_e_ | 0.691 | 0.790 | 0.759 | 0.806 | NA | 0.756 | 0.806 | 0.698 | 0.629 |
| H_O_ | 0.444 | 0.469 | 0.566 | 0.333 | NA | 0.557 | 0.706 | 0.639 | 0.579 |
| *F*_IS_ | 0.359** | 0.410** | 0.256** | 0.588** | NA | 0.264** | 0.127 | 0.085 | 0.081 |
| **Lid1** |  |  |  |  |  |  |  |  |  |
| N_A_ | 9.000 | 7.000 | 11.000 | 17.000 | 6.000 | 7.000 | 12.000 | 7.000 | 7.000 |
| A_R_ | 5.371 | 4.628 | 7.204 | 8.080 | 4.845 | 4.829 | 10.035 | 5.642 | 6.159 |
| H_e_ | 0.626 | 0.604 | 0.800 | 0.796 | 0.638 | 0.633 | 0.850 | 0.743 | 0.597 |
| H_O_ | 0.577 | 0.559 | 0.547 | 0.662 | 0.656 | 0.576 | 0.882 | 0.583 | 0.550 |
| *F*_IS_ | 0.079 | 0.076 | 0.318** | 0.170 | -0.028 | 0.090 | -0.039 | 0.217 | 0.081 |
| **CypG3** |  |  |  |  |  |  |  |  |  |
| N_A_ | 17.000 | 11.000 | 16.000 | 18.000 | 10.000 | 16.000 | 9.000 | 9.000 | 9.000 |
| A_R_ | 7.111 | 7.157 | 7.259 | 8.148 | 6.589 | 8.336 | 7.500 | 7.626 | 6.292 |
| H_e_ | 0.582 | 0.545 | 0.583 | 0.706 | 0.542 | 0.692 | 0.778 | 0.607 | 0.440 |
| H_O_ | 0.579 | 0.552 | 0.569 | 0.530 | 0.563 | 0.588 | 0.375 | 0.500 | 0.450 |
| *F*_IS_ | 0.005 | -0.012 | 0.024 | 0.251 | -0.038 | 0.151 | 0.526** | 0.181 | -0.024 |
| **CypG48** |  |  |  |  |  |  |  |  |  |
| N_A_ | 16.000 | 11.000 | 13.000 | 19.000 | 10.000 | 14.000 | NA | 11.000 | 9.000 |
| A_R_ | 9.684 | 8.795 | 10.405 | 11.278 | 8.208 | 9.486 | NA | 8.339 | 8.425 |
| H_e_ | 0.884 | 0.885 | 0.874 | 0.910 | 0.861 | 0.891 | NA | 0.862 | 0.847 |
| H_O_ | 0.861 | 0.912 | 0.850 | 0.846 | 0.765 | 0.774 | NA | 0.778 | 0.786 |
| *F*_IS_ | 0.026 | -0.031 | 0.029 | 0.071 | 0.113 | 0.133 | NA | 0.099 | 0.074 |
| **Ca1** |  |  |  |  |  |  |  |  |  |
| N_A_ | 9.000 | 6.000 | 11.000 | 13.000 | 10.000 | 12.000 | 5.000 | 8.000 | 8.000 |
| A_R_ | 6.410 | 4.919 | 7.010 | 7.072 | 8.023 | 7.541 | 4.961 | 5.925 | 6.635 |
| H_e_ | 0.779 | 0.724 | 0.816 | 0.789 | 0.775 | 0.831 | 0.759 | 0.765 | 0.744 |
| H_O_ | 0.605 | 0.588 | 0.528 | 0.574 | 0.758 | 0.690 | 0.600 | 0.618 | 0.579 |
| *F*_IS_ | 0.224** | 0.190 | 0.355** | 0.274** | 0.023 | 0.171** | 0.215 | 0.195 | 0.227 |
| **Ca3** |  |  |  |  |  |  |  |  |  |
| N_A_ | 20.000 | 15.000 | 23.000 | 22.000 | 17.000 | 19.000 | 13.000 | 18.000 | 14.000 |
| A_R_ | 11.712 | 11.588 | 12.749 | 13.681 | 12.687 | 12.945 | 12.347 | 11.545 | 11.704 |
| H_e_ | 0.919 | 0.919 | 0.928 | 0.940 | 0.933 | 0.938 | 0.939 | 0.905 | 0.914 |
| H_O_ | 0.754 | 0.783 | 0.873 | 0.721 | 0.800 | 0.721 | 0.929 | 0.750 | 0.778 |
| *F*_IS_ | 0.181 | 0.151 | 0.060 | 0.234** | 0.145 | 0.234** | 0.012 | 0.174 | 0.153 |
| **Ca12** |  |  |  |  |  |  |  |  |  |
| N_A_ | 21.000 | 17.000 | 17.000 | 23.000 | 14.000 | 16.000 | 8.000 | 16.000 | 8.000 |
| A_R_ | 10.383 | 11.473 | 10.956 | 10.921 | 8.767 | 9.375 | 7.016 | 10.661 | 6.337 |
| H_e_ | 0.860 | 0.907 | 0.886 | 0.894 | 0.846 | 0.803 | 0.761 | 0.856 | 0.704 |
| H_O_ | 0.785 | 0.824 | 0.807 | 0.700 | 0.824 | 0.767 | 0.588 | 0.774 | 0.500 |
| *F*_IS_ | 0.088 | 0.094 | 0.090 | 0.218** | 0.027 | 0.045 | 0.233 | 0.097 | 0.295 |
| **Lc27** |  |  |  |  |  |  |  |  |  |
| N_A_ | 5.000 | 2.000 | 5.000 | 5.000 | 4.000 | 3.000 | 2.000 | 2.000 | 4.000 |
| A_R_ | 2.290 | 1.991 | 2.836 | 2.595 | 2.442 | 2.199 | 1.998 | 1.960 | 3.853 |
| H_e_ | 0.135 | 0.255 | 0.208 | 0.232 | 0.141 | 0.136 | 0.226 | 0.187 | 0.477 |
| H_O_ | 0.141 | 0.235 | 0.190 | 0.214 | 0.147 | 0.127 | 0.250 | 0.206 | 0.474 |
| *F*_IS_ | -0.048 | 0.077 | 0.091 | 0.077** | -0.041 | 0.067 | -0.111 | -0.100 | 0.006 |
| **Lc290** |  |  |  |  |  |  |  |  |  |
| N_A_ | 7.000 | 7.000 | 9.000 | 9.000 | 7.000 | 10.000 | 9.000 | 10.000 | 8.000 |
| A_R_ | 5.879 | 5.431 | 6.570 | 7.215 | 5.563 | 6.608 | 8.188 | 7.161 | 6.968 |
| H_e_ | 0.758 | 0.720 | 0.779 | 0.843 | 0.755 | 0.776 | 0.863 | 0.839 | 0.827 |
| H_O_ | 0.695 | 0.559 | 0.667 | 0.716 | 0.742 | 0.794 | 0.813 | 0.583 | 0.667 |
| *F*_IS_ | 0.084** | 0.226 | 0.145** | 0.151** | 0.018 | -0.022 | 0.060 | 0.308 | 0.198 |
| **Lco4** |  |  |  |  |  |  |  |  |  |
| N_A_ | 5.000 | 3.000 | 5.000 | 10.000 | 3.000 | 5.000 | 6.000 | 5.000 | 6.000 |
| A_R_ | 3.657 | 2.896 | 3.724 | 5.541 | 2.833 | 3.666 | 5.601 | 4.177 | 5.143 |
| H_e_ | 0.586 | 0.525 | 0.565 | 0.728 | 0.551 | 0.590 | 0.740 | 0.606 | 0.704 |
| H_O_ | 0.549 | 0.529 | 0.466 | 0.522 | 0.588 | 0.516 | 0.529 | 0.270 | 0.700 |
| *F*_IS_ | 0.063 | -0.008 | 0.177 | 0.285** | -0.069 | 0.126 | 0.291 | 0.558** | 0.006 |
| **Mean** |  |  |  |  |  |  |  |  |  |
| N_A_ | 11.500 | 8.500 | 11.700 | 14.900 | 9.000 | 11.000 | 7.889 | 9.100 | 7.900 |
| A_R_ | 6.716 | 6.411 | 7.271 | 8.244 | 6.662 | 6.992 | 7.116 | 6.736 | 6.672 |
| H_e_ | 0.682 | 0.687 | 0.720 | 0.764 | 0.672 | 0.705 | 0.747 | 0.707 | 0.688 |
| H_O_ | 0.599 | 0.601 | 0.606 | 0.582 | 0.649 | 0.611 | 0.630 | 0.570 | 0.606 |
| *F*_IS_ | 0.122** | 0.128 | 0.159** | 0.240** | 0.034 | 0.134** | 0.160** | 0.196** | 0.122 |

Table S2. Individual locus-by-location estimates of genetic variation observed within the River Stour. N_A_ = the number of unique alleles, A_r_ = allelic richness, H_e_ = expected heterozygosity, H_o_ = observed heterozygosity, *F*_IS_ = population-level inbreeding coefficient (underlined values indicate a significant deviation of allele frequencies from those expected under a null model of Hardy-Weinberg equilibrium; values with * are significant before Bonferroni correction).

|  |  | Upstream | | | | | | | | | | | | |
| --- | --- | --- | --- | --- | --- | --- | --- | --- | --- | --- | --- | --- | --- | --- |
|  |  | From | | | | | | | | | | | | |
|  |  | MWP | DL | OW | CI | T | W | DO | DY | C | E | N | B | R |
| To | MWP | 0.8850 | 0.0171 | 0.0100 | 0.0091 | 0.0191 | 0.0072 | 0.0086 | 0.0071 | 0.0073 | 0.0073 | 0.0073 | 0.0071 | 0.0072 |
|  | DL | 0.0074 | 0.7470 | 0.1850 | 0.0091 | 0.0132 | 0.0072 | 0.0086 | 0.0042 | 0.0043 | 0.0042 | 0.0043 | 0.0043 | 0.0043 |
|  | OW | 0.0072 | 0.0047 | 0.8570 | 0.0161 | 0.0107 | 0.0054 | 0.0238 | 0.0055 | 0.0054 | 0.0054 | 0.0053 | 0.0054 | 0.0053 |
|  | CI | 0.0046 | 0.0565 | 0.1840 | 0.6920 | 0.0155 | 0.0041 | 0.0179 | 0.0041 | 0.0042 | 0.0042 | 0.0041 | 0.0042 | 0.0041 |
|  | T | 0.0067 | 0.0058 | 0.1850 | 0.0099 | 0.6750 | 0.0042 | 0.0880 | 0.0042 | 0.0043 | 0.0043 | 0.0043 | 0.0043 | 0.0042 |
|  | W | 0.0098 | 0.0091 | 0.0130 | 0.0096 | 0.0095 | 0.6760 | 0.2220 | 0.0083 | 0.0084 | 0.0084 | 0.0082 | 0.0083 | 0.0083 |
|  | DO | 0.0078 | 0.0148 | 0.0414 | 0.0089 | 0.0248 | 0.0074 | 0.8510 | 0.0074 | 0.0071 | 0.0074 | 0.0073 | 0.0072 | 0.0072 |
|  | DY | 0.0073 | 0.0121 | 0.0192 | 0.0067 | 0.2330 | 0.0067 | 0.0074 | 0.6740 | 0.0065 | 0.0066 | 0.0067 | 0.0066 | 0.0064 |
|  | C | 0.0061 | 0.0087 | 0.0196 | 0.0062 | 0.0072 | 0.0057 | 0.2450 | 0.0057 | 0.6730 | 0.0058 | 0.0058 | 0.0058 | 0.0057 |
|  | E | 0.0067 | 0.0259 | 0.2160 | 0.0073 | 0.0147 | 0.0065 | 0.0147 | 0.0066 | 0.0067 | 0.6740 | 0.0067 | 0.0067 | 0.0065 |
|  | N | 0.0082 | 0.0105 | 0.0320 | 0.0082 | 0.0126 | 0.0079 | 0.2060 | 0.0077 | 0.0078 | 0.0079 | 0.6760 | 0.0075 | 0.0077 |
|  | B | 0.0076 | 0.0080 | 0.2190 | 0.0075 | 0.0098 | 0.0076 | 0.0273 | 0.0076 | 0.0075 | 0.0075 | 0.0077 | 0.6760 | 0.0076 |
|  | R | 0.0087 | 0.0103 | 0.0139 | 0.0087 | 0.0200 | 0.0085 | 0.2100 | 0.0085 | 0.0087 | 0.0086 | 0.0085 | 0.0085 | 0.6770 |

Table S3. BAYEASS+ estimates of migration rates among sub-populations in the River Thames. The proportional values in the diagonal cells represent non-migrants. Underlined values fall within the top 10% of estimates. Significant contributing sub-populations to the migrant pool are highlighted (net negative migration).

|  |  | Upstream | | | | | | | | |
| --- | --- | --- | --- | --- | --- | --- | --- | --- | --- | --- |
|  |  | From | | | | | | | | |
|  |  | BL | DM | ST | AB | SW | MM | RC | SbC | TH |
| To | BL | 0.9350 | 0.0035 | 0.0308 | 0.0035 | 0.0035 | 0.0182 | 0.0035 | 0.0035 | 0.0045 |
|  | DM | 0.2820 | 0.6740 | 0.0072 | 0.0062 | 0.0061 | 0.0064 | 0.0062 | 0.0061 | 0.0062 |
|  | ST | 0.2680 | 0.0035 | 0.6990 | 0.0034 | 0.0040 | 0.0120 | 0.0034 | 0.0034 | 0.0037 |
|  | AB | 0.2760 | 0.0046 | 0.0091 | 0.6720 | 0.0046 | 0.0077 | 0.0046 | 0.0046 | 0.0175 |
|  | SW | 0.2960 | 0.0042 | 0.0062 | 0.0042 | 0.6710 | 0.0052 | 0.0042 | 0.0042 | 0.0046 |
|  | MM | 0.2770 | 0.0036 | 0.0208 | 0.0037 | 0.0036 | 0.6800 | 0.0037 | 0.0035 | 0.0040 |
|  | RC | 0.0167 | 0.0100 | 0.2430 | 0.0099 | 0.0098 | 0.0109 | 0.6790 | 0.0098 | 0.0104 |
|  | SbC | 0.0173 | 0.0059 | 0.2720 | 0.0058 | 0.0057 | 0.0080 | 0.0058 | 0.6730 | 0.0061 |
|  | TH | 0.0297 | 0.0152 | 0.0297 | 0.0156 | 0.0158 | 0.0175 | 0.0157 | 0.0143 | 0.8650 |

Table S4. BAYEASS+ estimates of migration rates among sub-populations in the River Stour. The proportional values in the diagonal cells represent non-migrants. Underlined values fall within the top 10% of estimates. Significant contributing sub-populations to the migrant pool are highlighted (net negative migration).


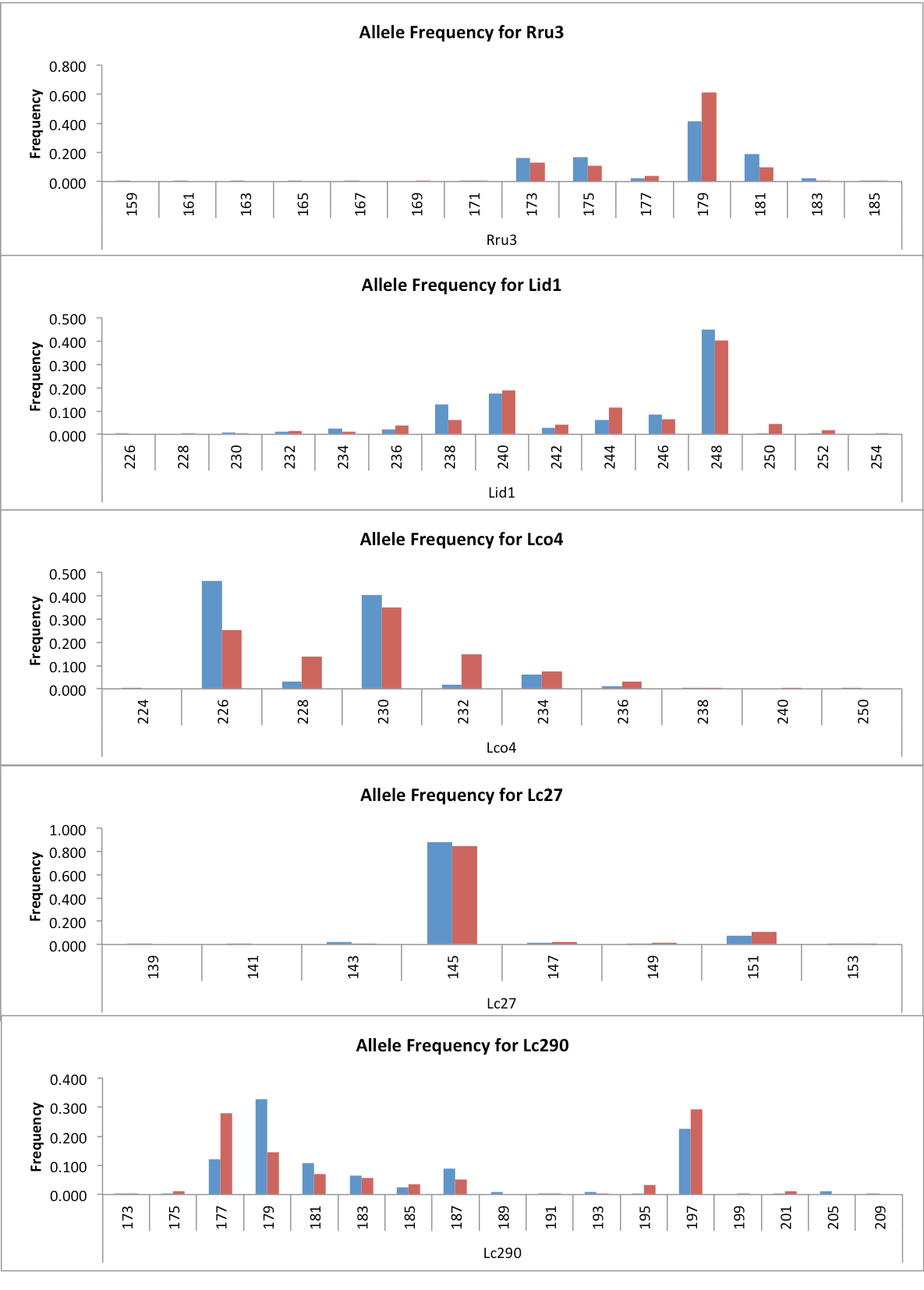


Fig S1a. Allele frequency histograms for each locus utilised in this study summed over each location for the Rivers Thames (red) and Stour (blue). Numbers indicate the number of repeat motifs in each.


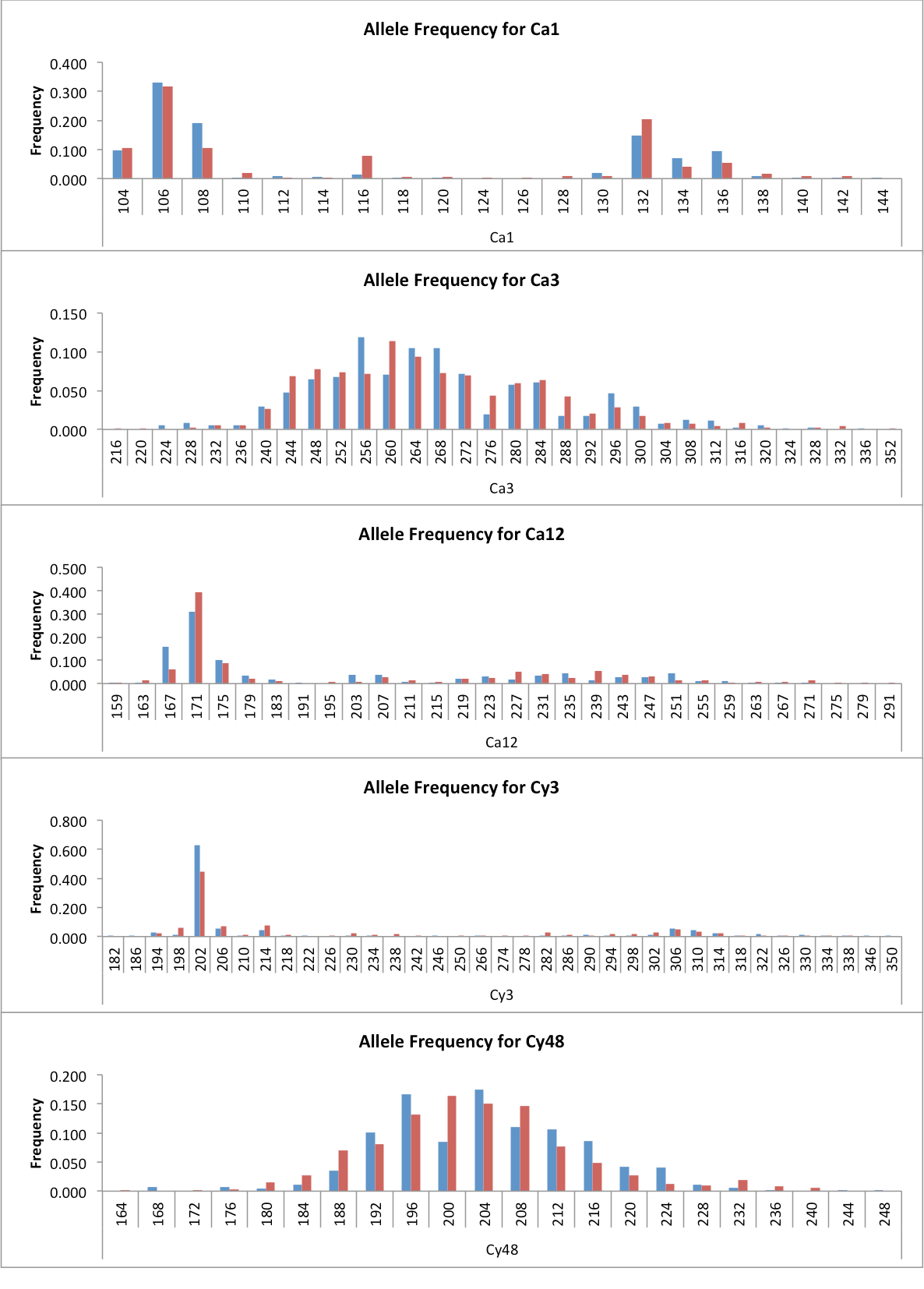


Fig S1b. Continuation of frequency data.

Fig S2. STRUCTURE analysis. We assessed cryptic population structure by adopting a Bayesian framework optimised in the program STRUCTURE 2.3.3 (Pritchard et al. 2000). This program uses allele frequencies to derive prior probabilities of the number of ‘true’ ‘K’ HWE populations into which individuals are clustered (K_1_, K_2_,…K_n_, where K_n_ is the nth whole number of populations). The program was run with and without prior information regarding the sampling location of individuals. Utilising sampling information was found to better inform prior distributions of K in instances of high within population diversity and low inter-population divergence (Hubisz et al. 2009). Both runs used an admixture model of inference and correlated allele frequencies to account for the recent evolutionary co-ancestry of populations (Falush et al. 2003). 20 values of K were analysed, with each analysis iterated 5 times (5x10^5^ MCMC iterations after 10^5^were discarded as burn-in). The optimal value of K was calculated according to the ΔK procedure (Evanno et al. 2005) in STRUCTURE HARVESTER (Earl and von Holdt 2011). The output was visualised in DISTRUCT version 1.1 (Rosenberg 2004). Top panel displays K = 2 populations when no geographic information was inputted into the Bayesian analysis. Lower panel displays K = 5 populations when geographic location information was incorporated as prior information in the Bayesian analysis. Each diagram shows the proportion of each inferred K populations’ genomic signal (Q) found in each individual (vertical lines) in each sub-population (shown in strong black outline).

Earl, D. A. & van Holdt, B. M. (2011). STRUCTURE HARVESTER: a website and program for visualizing STRUCTURE output and implementing the Evanno method. *Conservation Genetics Resources*, DOI:10.1007/s12686-011-9548-7.

Evanno, G., Regnaut, S. & Goudet, J. (2005) Detecting the number of clusters of individuals using the software STRUCTURE: a simulation study. *Mol Ecol*, **14**, 2611-2620.

Falush, D., Stephens, M. & Pritchard, J. (2003) Inference of population structure: extensions to linked loci and correlated allele frequencies. *Genetics*, **164**, 1567-1587.

Hubisz, M., Falush, D., Stephens, M. & Pritchard, J. (2009) Inferring weak population structure with the assistance of sample group information. *Mol Ecol Resourc*, **9**, 1322-1332.

Rosenberg, N. A. (2004) DISTRUCT: a program for the graphical display of population structure. *Mol Ecol Notes*, **4**, 127-138.

Fig S3. An example of Simpson’s Paradox. Both genetic distance (left hand axis, open triangles and hatched line of best-fit) and the number of weirs (right-hand axis, solid squares and unbroken line of best-fit) are significantly and positively correlated with genetic divergence (x-axis, *F*_ST_). However, when one environmental distance variable is regressed against genetic distance whilst controlling for the other, the correlation between the coefficients for the environmental distance variable becomes negative for one of the two comparisons (schematically illustrated by the arrow), in this case weirs and genetic
